# Emotional Well-Being Human Studies

**DOI:** 10.1093/geroni/igab046.780

**Published:** 2021-12-17

**Authors:** Feng Lin

**Affiliations:** Stanford University, California, United States

## Abstract

Early evidence indicates an association between EWB and underlying brain processes, and that those processes change with both normal and pathological brain aging. However, the nature of these associations, the mechanisms by which EWB and its component domains change with brain aging, and how those changes may be associated with common neuropathologies in ADRD, are largely unexplored. We propose an appraisal-adaptation model in understanding relationships between EWB and ADRD. For human models, we encourage the use of well-established measures that directly assess eudaimonic and hedonic EWB, including abnormal scenarios (e.g., neuropsychiatric symptoms, anhedonia, loneliness, etc.), as well as older adults with exceptional cognition (i.e., superagers or supernormals). Dr. Lin will review premises associated with the appraisal-adaptation model in conducting human research on EWB, aging, and ADRD.

